# The Institutional Treatment of Consumption

**Published:** 1916-07-01

**Authors:** 


### THE INSTITUTIONAL TREATMENT OF CONSUMPTION.

### V.—The Disposal of Sputum.*

Two very important items in the medical equipment of an institution for the treatment of tuberculous patients must next be considered. These are the sputum cups and flasks and the weighing machines. Nearly all the patients will require to use at some time of the day or night a receptacle in which to spit; some will only expectorate early in the morning, while the "hospital" case must have a sputum mug constantly at hand by the bedside. Those patients who are up and about are provided with a portable flask if they still expectorate, the use of mugs being confined to the wards on account of their liability to be upset. There are naturally many types of cups and flasks in use, but probably the most frequently met with cup is the enamelled round mug with a handle and a lid. The lid is swung on a projecting piece of metal, and is so constructed as to be readily raised by the thumb of the hand holding the mug; this is a great advantage, as may be imagined. The lid also will not remain open when released, nor can it easily fall off. These iron cups are not difficult to clean and to sterilise, and will last a long time even with hard usage.

Another form of cup is that made of compressed pulp, exactly similar to those familiar receptacles used in dairies for retailing cream. These are, of course, burnt after use. They have the serious disadvantage that they are easily upset by a light blow or by a puff of wind; this defect is remedied by placing them in holders, but this method of providing for the expectoration of patients is not an economical one. A cheaper method is to supply thin paper linings to the enamelled mugs; this simplifies the process of sterilisation, as tenacious sputum has not then to be poured out of the cups.

### The Design of Sputum Flasks.

Of sputum flasks which patients carry about with them the most familiar is the flat oval of blue glass with a wide mouthpiece covered by a cap. Some authorities supply spittoons with a rubber cork—an unsafe form of stopper. With the object of assisting the process of keeping these flasks clean and sweet some are made with an opening at the bottom. This is a form to be preferred, as it has the advantage of being able to be washed right through. It is important, however, that the cap closing the bottom opening should be capable of being firmly closed and not likely to allow of the slightest oozing. Dettweiler's flask is made on this pattern, but in addition the upper opening is prolonged inside on the principle of the unspillable inkpot. This pattern is too expensive for working-class sanatoria, and is rather complicated. Simpler forms are readily obtainable in the market.

It is usual to place in sputum cups a little liquid antiseptic; 1 in 20 carbolic is commonly used, as it is clear and does not interfere with the doctor's inspection of the expectoration; but other fluids may be used on the score of economy without much disadvantage. In fact, plain water alone may be placed in the cups. For the flasks a little antiseptic is commonly ordered, but a good plan is to place in these pocket spittoons a few crystals of common washing soda. With this the sputum will form a jelly-like mass which is not likely to leak through a faulty cap and is easily turned out of the flask when the patient gets back to the ward or to his home.

In institutions the disposal of the sputum day by day is a problem which calls for special arrangements. In most cases sterilisation in a special steam-heated apparatus is the preferred method. The ward cups and the portable spittoons must be treated separately. In the case of the former the contents may either first be emptied into a cardboard or wooden box full of sawdust or the cups may be transported just as they come from the bedside to the disinfecting plant. In large sanatoria the cups may have to be conveyed over bumpy paths for a considerable distance, and the risk of some of the contents being spilt is not a small one; hence the suggestion of first emptying them, though just at present this is an expensive method owing to the cost of wood and paper.

### Principles of Disinfection.

In dealing with the disinfection of sputum it is very desirable that a method should be adopted which reduces the handling of cups and flasks to a minimum, and thus the risk of infection to the attendant. Hence it is common to place the cups just as they are collected into a special cauldron containing a solution of soda, which is brought to the boil by means of steam. The mixture of sputum and soda solution, after being boiled for twenty minutes, is run off and the sterilised pots thoroughly rinsed in fresh water. Glass spittoons are treated similarly, but are placed first in cold soda solution, their caps being removed and thrown in too; heat is applied more gradually in order to avoid cracking the glass, but otherwise the method is similar.

In connection with the subject of expectoration it should be mentioned that in sanatoria it is one of the deadly sins for a patient to use an ordinary handkerchief and to keep this tucked under the pillow or in a coat pocket. The danger of such a habit is obvious, and no such incubator and disseminator of tubercle bacilli can be allowed. Hence patients are provided with "handkerchiefs," usually of butter-muslin, for use while resident. Bed patients are instructed to keep these handkerchiefs in special receptacles on their lockers—a tin mug would do; ambulant patients are provided with washable pocket linings in which to carry these handkerchiefs—women usually pin

* The previous articles appeared on May 6 and 20, and June 3 and 17, 1916.

these pockets on to their skirts. The butter-muslin may be replaced by Japanese paper napkins, and in either case the handkerchiefs are frequently renewed, the used ones being burnt. Small pieces are all that is necessary, for they are only intended for wiping the mouth after expectorating. Here it may be observed that male patients should be persuaded to shave off beard and moustache; it is impossible otherwise to avoid germ-laden sputum from drying on the lips or chin.

Among the signs of favourable progress a good deal of importance is attached, particularly by the patient himself, to an increase of weight. The usual weekly weighings are occasions of interest and some excitement. Other signs of satisfactory progress are to the physician of more value, but a record of the patient's weight is certainly one of the last things which would be omitted. Patients of the hospital type with advancing disease may put on weight which has then no favourable prognostic significance, but among the sanatorium cases an increase of weight up to a reasonable point is an encouraging sign, when temperature and the general condition are satisfactory. It is therefore a matter of importance to provide each hospital and sanatorium block with an efficient weighing machine. It is a very poor plan to have to carry machines from ward to ward, so that a sufficient number should be ordered to avoid this. Of the type of machine to be recommended it is unnecessary to say more than that in hospital wards the machine should be of the kind which provides a chair for the patient while being weighed. In sanatorium blocks, too, it is just as well to have this kind unless the additional expense is thought to be too great. Patients are weighed with a minimum of clothing, and care must be taken that the coverings of the body and feet are always the same at each weighing.

The patients approach in turn suitably prepared, and either a medical officer or a nurse does the actual manipulation of the weights. The other official gets ready the appropriate history sheet and, to facilitate the weighing, reads out the weight of the patient last time the record was made. The new weight is entered on the sheet at once, to avoid any errors of copying from one slip of paper to another.

